# Mapping environmental perceptions in Romania: A mixed-methods research

**DOI:** 10.1016/j.heliyon.2024.e40845

**Published:** 2024-11-29

**Authors:** Ruxandra Malina Petrescu-Mag, Adrian Ivan, Cornel Pantelimon, Dacinia Crina Petrescu

**Affiliations:** a Department of Environmental Science, Faculty of Environmental Science and Engineering, Babes-Bolyai University, 30 Fantanele Street, Cluj-Napoca 400294, Romania; b Doctoral School “International Relations and Security Studies”, Babes-Bolyai University, 1 Mihail Kogalniceanu Street, Cluj-Napoca, 400084, Romania; c Department of Economy and Rural Development, Faculty of Gembloux Agro-Bio Tech, University of Liège, Passage des Déportés, 2, 5030, Gembloux, Belgium; d Department of International Studies and Contemporary History, Faculty of History and Philosophy, Babes-Bolyai University, Cluj-Napoca, Romania; e Department of Hospitality Services, Faculty of Business, Babes-Bolyai University, 7 Horea Street, Cluj-Napoca 400174, Romania

**Keywords:** empathy map, Values, Behaviors, Motivations, Attitude, Nature, Interviews, Bibliometric method

## Abstract

The present study investigates environmental perceptions in Romania, emphasizing their role in shaping individual and collective responses to ecological challenges. By exploring how people understand and interact with their natural environment, the research aims to explore values, needs, behaviors, and motivations that drive pro-environmental actions. We used a mixed-methodology approach, combining qualitative and quantitative analyses to capture a comprehensive view of these perceptions. An innovative empathy map was developed through one-on-one interviews to visualize and analyze participants' perceptions of the environment. Participants were characterized by a biocentric-anthropocentric value orientation, balancing respect for nature with human needs. They considered that the top three environmental problems in Romania were: water and air pollution, deforestation, and poor waste management. Complementing this, a bibliometric analysis was conducted to examine the co-occurrence of keywords related to environmental perceptions in the academic literature, providing a quantitative perspective that aligned with the themes from the qualitative analysis. The strong presence of terms like “intention” and “energy consumption” in the analysis of keywords co-occurrence supported the idea that personal and social norms around energy use were significant themes in the environmental literature dedicated to Romania, which were also central to the participants’ perceptions. The findings contribute to a deeper understanding of environmental perceptions in Romania, offering useful insights for policymakers and environmental managers to promote sustainable development and pro-environmental behaviors in the region.

## Highlights

• Environmental perceptions in Romania were investigated.
• A mixed method was used: interviews and bibliometric analysis.
• An empathy map was created, presenting values, needs, behaviors, motivations, and obstacles related to the environment.
• Participants had a biocentric-anthropocentric value orientation.
• The findings help policymakers and environmental managers promote sustainability and pro-environmental behaviors.

## 1 Introduction

Understanding environmental perceptions is fundamental to addressing contemporary ecological challenges. Environmental perceptions shape how individuals and communities understand and interact with their surroundings [1,2], guiding behaviors, shaping attitudes toward conservation efforts, and impacting decisions related to sustainable practices [3,4]. Research has shown that these perceptions can drive pro-environmental behaviors, such as recycling and energy conservation, while also affecting support for environmental policies and community-led initiatives [[5], [6], [7]]. In this study, “environmental perception” is defined as the way individuals observe, interpret, and evaluate the natural environment and related experiences, policies, or outcomes [8]. This meaning is similar to the one used in other qualitative research [9], and not identical to that of “perception” defined by psychologists as the process of receiving, processing, and understanding sensory information. Bennett’s [8] definition reflects the broader meaning of “perception,” often used in social sciences, which is distinguished from more specific concepts like beliefs or attitudes [8,[10], [11], [12]]. For example, Ajzen [13] considers attitude as people’s evaluations of an object, person, or action. Following Ajzen’s definition, certain answers of this study can be considered as reflecting people’s attitudes, while this study classified them generally as perceptions. We did this because this study presents interviewed people’s subjective perspectives and descriptions of how they perceived (understood) aspects (e.g., values and behaviors) about others and themselves. Consequently, “perception” is used here as an umbrella term encompassing participants' *understandings* and *ways of viewing* their or others' natural environment, values, needs, behavior, etc. Environmental perception is inherently subjective and shaped by social and cultural factors, and an individual’s background, history, and surrounding environment [8]. Perceptions are closely intertwined with values, attitudes, and norms, playing a crucial role in shaping behaviors, motivations, and the level of support for various pro-environmental initiatives [14]. Unlike more narrowly defined terms, environmental perception encompasses a person’s overall understanding and interpretation of the environment, making it a key factor in how individuals and communities interact with and respond to environmental challenges. This broader definition allows for capturing a wide range of influences and interpretations that shape people’s relationship with the environment, setting this study’s approach apart from others focusing specifically on attitudes or beliefs alone.

Given the strong connection between perceptions and the underlying values, attitudes, and norms, it is not surprising that environmental perceptions can vary significantly across different cultural and geographic contexts. A rich vein of studies examined environmental perceptions on a global scale [2,15,16], indicating significant variations in environmental perceptions across different countries [[17], [18], [19]]. These variations highlight the importance of analyzing environmental perceptions within specific regional and national contexts for a more nuanced understanding. For example, in Romania, such perceptions are shaped by a combination of historical, cultural, and socio-economic factors [20,21].

At the same time, the transition toward more sustainable behaviors faces challenges, such as inconsistent environmental education and competing economic priorities [22,23]. Given these dynamics, understanding how Romanians perceive the state of the environment is relevant for developing tailored policies and interventions that address their specific concerns, values, and behaviors and identify predictors of pro-environmental actions. Here, the concept of “environment” refers specifically to the natural environment, considering all its components – air, water, plant and animal species, land, and landscapes.

While it is essential to grasp these broader perceptions, much of the existing research has focused on specific components of the natural environment, providing valuable insights into how Romanians view environmental components or actions related to them, like air quality, land use, and water management. Previous research dedicated to the Romanians' perception of the natural environment investigated specific environmental components, such as air [24], land [25], water [26,27], and forest [28,29]. Additionally, other studies focused on environmental problems or challenges, such as climate change [[30], [31], [32], [33]], ecosystem services provided by a particular environmental component, such as a river or mountain [34,35], or human-to-nature relationships [20,36]. One study [37] used the phrase “environmental perception” but it referred to aspects different than those envisaged in the present analysis. In the cited paper, this concept referred to satisfaction with specific objects and places from the surrounding living environment (e.g., things from the house, the yard of the household, the street they live on, and their garden). In the present paper, environmental perceptions encompass perceptions about the natural environment as a whole, with all its components (air, water, etc.). In this context, and to the authors’ best knowledge, no studies have specifically addressed the general environmental perceptions of Romanian people. Addressing this gap is essential, as a broader understanding of local environmental perceptions can provide valuable insights into how communities interact with their surroundings.

Investigating local perceptions of the environment offers several significant advantages, as previously highlighted in other research [8,38]. For instance, understanding these perceptions is crucial to the success of conservation initiatives. This is because local perceptions, although subjective, represent a vital aspect of reality for individuals and communities, influencing their support for or opposition to restoration and conservation measures [39]. Moreover, understanding public perception allows environmental managers to identify the underlying reasons for the lack of support and implement targeted interventions to foster long-term commitment to conservation goals [40]. Furthermore, investigating local people’s perception of environmental and human-nature connectedness is indispensable to achieving sustainability [41,42]. Additionally, insights gained from research on local perceptions can be integrated into environmental decision-making and co-management [43].

### 1.1 Research objectives and exploratory and research questions

While previous studies addressed various components of the natural environment in Romania, a comprehensive exploration of general environmental perceptions remains underexplored. To fill this gap, this study seeks to deepen insights by mapping participants’ values, needs, and behaviors concerning the environment and motivations and obstacles related to these behaviors. In pursuit of this goal, it employs an empathy map and analyzes the co-occurrence of keywords concerning these perceptions. This dual approach allows for a nuanced understanding of how these perceptions are embedded within the broader academic landscape dedicated to Romania. Consequently, two objectives of the study were set:

i) To explore and visualize in an empathy map the values, needs, behaviors, motivations, and obstacles of participants regarding the state of the environment, and
ii) To identify, analyze, and visualize the co-occurrence of keywords related to environmental perceptions, specifically reflecting and interconnecting the deductive and inductive codes derived from the thematic analysis of the empathy map in the existing literature on Romania.

To fulfill the research objectives, the present study adopted a mixed methodology to leverage the strengths of both qualitative and quantitative approaches. Qualitative research allows for an in-depth and contextual exploration of participant experiences and perspectives, capturing nuanced insights that might be overlooked by numerical data alone [44]. Based on one-to-one interviews, we developed an empathy map [after 45,46]. We used the empathy map as a visualization tool to gain a deeper understanding of participants’ behavior, values, motivations, and obstacles by emphasizing their perspective on the world [46,47] – specifically, their views on the state of the environment. Qualitative research answered the following exploratory questions (EQ) that reflected the components of the empathy map:

**EQ (1). Think & Feel:** “What do I feel and what do I think about the environment?” (It focuses on values and needs related to the environment – what happens in the respondent’s mind).

**EQ (2). See:** “What do I see related to protecting the environment?” (It is about third parties’ behavioral patterns – what the respondent sees in his/her environment).

**EQ (3). Say & Do:** “What do I say and do for the environment?” (It focuses on the respondent’s behavior toward the environment, obstacles, and motivations – what the respondent says and how s/he behaves).

**EQ (4). Hear:** “What do I hear about environmental protection?” (It focuses on the sources of information and influence – how the others influence the respondent).

Next, the analysis of keywords co-occurrence was considered relevant as a quantitative approach. This was used to map the research field of environmental perceptions in Romania. The keywords used for this purpose were, in their majority, the key themes revealed by the thematic analysis performed in the previous research stage (qualitative). The corresponding research question was formulated as follows: **RQ.** “How are the deductive and inductive codes derived from the thematic analysis of the empathy map reflected and interconnected in the existing literature?”

While the thematic analysis provides deep qualitative insights into respondents’ perceptions of the environment, the bibliometric analysis complements it by offering a quantitative perspective on how these perceptions are reflected and interconnected in the academic literature.

To the authors' knowledge, this study is the first to use an empathy map to visualize and deeply understand participants' needs, values, behaviors, or motivations regarding the environment. This innovative approach offers a comprehensive view of Romanian perceptions of the environment, a relatively underexplored topic. While the qualitative analysis provides contextual insights into participants’ perceptions, the bibliometric analysis fed in the Web of Science Core Collection can validate whether these insights are consistent with or divergent from broader academic discourse.

Practically, this combined approach ensured that both the micro-level qualitative insights and macro-level bibliographic trends were addressed, offering a holistic view of environmental perceptions in Romania. In essence, the qualitative data provided a rich, grounded understanding of participants’ perceptions, while the quantitative analysis offered a way to generalize these insights and identify their place within the broader field of study. This integration ensured that our conclusions reflected both the specific, lived experiences of the participants and the wider academic context, providing a holistic view of environmental perceptions in Romania. Furthermore, by blending detailed qualitative insights with quantitative data, the study provided a nuanced understanding of environmental perceptions in Romania, enhancing the validity and robustness of the conclusions.

## 2 Methodology

### 2.1 Qualitative research

#### 2.1.1 The development of the interview script and the implementation of one-to-one interviews

The present study followed the COREQ guidelines, Consolidated Criteria for Reporting Qualitative Research [48]. Ethical approval for research development was obtained from The Scientific Council of the Babes-Bolyai University, Cluj-Napoca, Romania, no.1148/2/26.01.2024. The participants were informed about the research objective. Their anonymity was protected by assigning each participant a unique identifier using the letter “P” followed by a number (e.g., P1, P2), ensuring that their names, personal details, or the workplace were not linked to their responses. Confidentiality was maintained throughout the study, with all identifying information removed from the data. Participants’ involvement was entirely voluntary. Participants provided a written informed consent form before participating in the study.

**Fig. 1:**
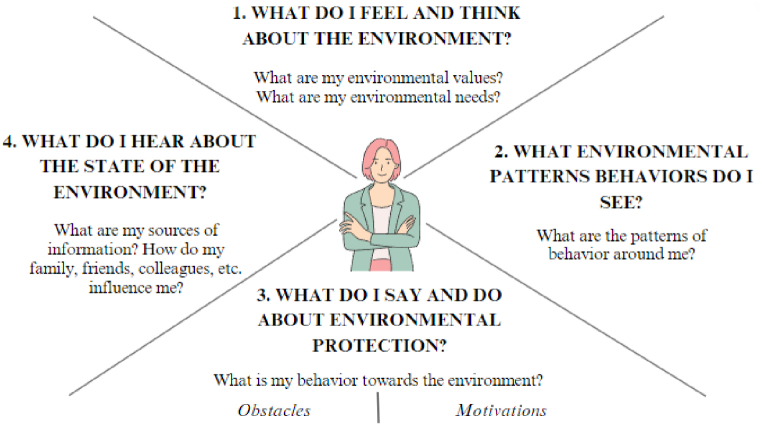
The empathy map: the four components that define its structure in the study and the main associated questions. Sources: adapted after [45,46].

Note: The questions included in the empathy map model [45,46] were highlighted in bigger fonts and capitalized; the questions created by the authors to reflect the questions of the model were mentioned in smaller fonts.

The interview script (presented in Table A1, Annex) included questions aimed at developing an empathy map that captured perceptions of the environment. Typically used to design business models based on customer perspectives, empathy maps have also found applications in various fields, mainly medical education and communication [49,50]. Usually, the empathy map has been designed with different components (four components [51], and six components [45,46]). The questions were divided into four main categories (“What do I feel and think?”, “What do I see?”, “What do I say and do?”, and “What do I hear?”), see Fig. 1.

The one-to-one interview method was chosen to elicit the participants' perceptions regarding the four selected components. We employed a semi-structured interview with a preestablished interview guide where the interviewer could ask follow-up questions and explored topics that appeared during the conversation. The interviews were conducted online and face-to-face, lasting between 45 and 60 min. The data collection took place from January to June 2024. All interviews (both the face-to-face and online ones) were audio recorded, and subsequently, the interviews were transcribed verbatim. Next, we conducted an intelligent transcription. Intelligent transcription means transcribing every word while interpreting the content to exclude pauses, filler words, and non-verbal sounds, and potentially improve grammar. We chose intelligent transcription over verbatim because it allowed us to focus on the main themes and insights rather than every verbal detail. Verbatim transcription included all speech patterns, which could be distracting when the goal was to analyze content rather than language use. Intelligent transcription provided a clearer, more readable dataset [52], making thematic analysis more efficient while accurately capturing participants’ views. The text resulting from this intelligent transcription in Romanian was used for the thematic analysis.

The participant selection method was the “snowball sampling” technique. This approach is popular in qualitative research [53,54]. The method began with a small group of initial participants (“seeds”) who were recruited for the study based on specific criteria (residential environment, education, gender). The initial participants were approached through direct outreach. We contacted participants through personal and professional networks (e.g., people involved in charity, animal welfare activities/campaigns, business associations, student associations, and sports clubs). The interviewer explained to them the study’s objectives and emphasized the importance of their contributions to understanding the broader context. Once the initial participants agreed to participate, they were asked to recommend others for the interview, thereby initiating the snowball sampling process. These new individuals were then contacted and invited to participate, and the process continued until data saturation was reached [55], which means no new significant information emerged. We reached saturation at the 34th interview. This method allowed us to gradually build a sample based on relationships and connections, thus gaining a deeper understanding of the social and cultural context of the participants [53].

Participants had to be at least 18 years old (the age of majority in Romania) to be eligible for the study, while those younger than 18 were excluded. From a demographic perspective, the average participant age was 38 years (with an age range between 21 and 67 years), 55.8 % were women, 64.7 % resided in urban areas, 82 % graduated higher education, 52.9 % reported a monthly family income exceeding 7001 lei (around 1400 Euro), 55.8 % had children, and 11.7 % reported suffering from chronic diseases. The total number of participants was 34. Collecting data on chronic illnesses was particularly relevant, as health status could significantly impact how individuals perceive and prioritize environmental issues.

The snowball sampling method presents challenges, such as subjectivity, as participants tend to recommend individuals like themselves or the influence of contextual variables on behaviors [56]. Additionally, dependence on the social networks of the initial participants can lead to an uneven representation of the studied population [57] citing [58]. This method can introduce biases, such as overrepresenting certain demographic groups or social characteristics, potentially affecting the diversity and representativeness of the sample.

In response to the risks brought by these limits, we took measures to promote sample diversity, as this is usually considered desirable, following Kirchherr & Charles [57]. Thus, we ensured the diversity of the sample seed (meaning that we began the sample with seeds that were as diverse as possible), including participants from both rural and urban areas to capture a range of geographic perspectives. We also selected seeds with varying ages, from young adults to older individuals, to avoid age-related biases and ensure that different life stages were represented. Furthermore, we usually repeated contacts/requests two times to obtain interview acceptance. This approach helped to minimize self-selection bias, where only those most eager or available might dominate the sample, ensuring a broader and more balanced representation of the population. In addition, we tried to include participants from diverse socioeconomic backgrounds, further enhancing the diversity of the starting sample. We also paid careful attention to gender representation, aiming to include a similar number of men and women, ensuring a balanced perspective across genders.

#### 2.1.2 Data analysis

Data analysis followed the following steps. 1) Transcription of interviews: All interviews were transcribed verbatim to ensure data accuracy and followed by intelligent transcription; 2) Initial coding: We used deductive codes based on the interview guide; c) Code review: Two of the authors reviewed and adjusted the codes as new themes emerged; d) Final coding: Two of the authors applied inductive codes; e) Data interpretation: Two of the authors analyzed the identified themes to extract conclusions relevant for the research objective.

In detail, the thematic analysis of interviews began with establishing deductive codes. These codes were extracted from the scientific literature [45,46,[59], [60], [61], [62], [63], [64]] (more details about the codes were included in Table A2, in the “3. Results” section, as they were considered part of the study results). Each interview transcript was systematically scrutinized, particularly for instances aligning with the predefined deductive codes. This initial coding phase ensured a comprehensive exploration of the predetermined themes and facilitated the organization of data according to established four main categories (“What do I feel and think?”, “What do I see?”, “What do I say and do?”, and “What do I hear?”). Subsequently, an inductive coding approach was employed to capture emergent themes and nuances not covered by the deductive framework. Through iterative examination of the data, additional codes were generated to accommodate new insights and unexpected patterns observed across interviews. The inductive process allowed for a flexible and exploratory analysis, enabling the identification of themes that were not anticipated as *a priori*. Integrating the deductive and inductive coding strategies facilitated a holistic understanding of the interview data, thus enriching the depth of the thematic analysis.

### 2.2 Quantitative research

#### 2.2.1 Bibliometric extraction and VOSviewer analysis

The Web of Science Core Collection (accessed through the Enformation web platform) was used to download the documents for VOSviewer analysis. We used only this database due to its quality and coverage. The Web of Science (WoS) is renowned for its rigorous selection process, ensuring high-quality, peer-reviewed content, and indexes almost all the important research papers [65]. It includes a wide range of disciplines and is known for its comprehensive indexing of influential journals. This quality control ensures that the data extracted are reliable and relevant for scientific analysis.

The particularized Boolean search was structured to retrieve all documents that met specific criteria related to environmental perceptions in Romania (see Table 1). The keywords were selected to align with the four empathy map components, and most of them were chosen from the deductive and inductive codes illustrated in Table A2. In addition to these codes, we used other keywords that we considered relevant (e.g., “eco-consciousness”, and “environmental ethics”). Consequently, to determine the final set of keywords, we used a combination of deductive and inductive codes (from Table A2). Deductive codes were derived from established theoretical frameworks and literature on environmental awareness and behaviors, ensuring that well-known concepts were included. Inductive codes, on the other hand, emerged from the interviews. Including all deductive and inductive codes from Table A2 would have resulted in an unmanageable dataset of papers, so we carefully balanced comprehensiveness with relevance. Therefore, we prioritized terms frequently appearing in initial searches, as they indicated significance within the existing body of literature. Conversely, we excluded overly broad terms that generated many unrelated results. For instance, while “environment” is a central keyword, it was too generic to capture the specific nuances we sought. Hence, we refined it into terms like “environmental awareness” or “environmental communication” to ensure precision and maintain a clear focus on themes relevant to environmental perceptions in Romania.

**Table 1:** Summary of bibliographic extraction parameters.

Bibliographic extraction				
Components of the empathy map	Think & Feel	See	Say & Do	Hear
**Keywords associated with the components of the empathy map (some of the deductive and inductive codes, and other words)**	(ALL=(“environmental values” OR “environmental awareness” OR “nature connectedness” OR “environmental concern” OR “environment∗ needs” OR “environmental attitudes” OR “eco-consciousness” OR “environmental ethics” OR “environmental sustainability values” OR “emotional response to environment∗)) AND ALL=(Romania)	ALL=(“environmental behavior’ OR “behavioral patterns” OR “environmental practices” OR “sustainable practices” OR “positive behavior” OR “negative behavior”)) AND ALL=(Romania) (ALL=(“economic factors” OR “time constraints” OR “convenience” OR “lack of infrastructure”)) AND ALL=(Romania and “environmental protection”)	ALL=(“personal health and well-being” OR “personal health” OR “concern for the self”)) AND ALL=(Romania and “environmental protection”) (no return) (ALL=(“pro-environmental activities” OR “community involvement” OR “consumer choices” OR “lifestyle changes” OR “eco-friendly practices” OR “green habits” OR “lack of awareness” OR “cost constraints” OR “budget constraints” OR “infrastructure accessibility" OR “time constraints” OR “barriers to sustainable behavior” OR “challenges in adopting green practices” OR “financial rewards” OR “economic incentives” OR "motivation for green behavior”)) AND ALL= (Romania and “environmental protection”)	ALL= (“environmental communication” OR “environmental awareness campaigns” OR “media coverage of environment∗” OR “public debate∗ on environment∗” OR “environmental narratives” OR “influence of environmental organizations” OR “environmental education” OR “environmental information sources” OR “peer influence on environm∗” OR “public opinion on environment∗” OR “social media influence on environment∗”) AND ALL= (Romania)
**Database**	Web of Science Core Collection			
**Type of selected document**	Article, proceeding paper, review article, book chapter, early access, editorial material, meeting abstract, and book review.			
**Other criteria**	All papers were in English; Region/country: Romania; Timeframe: No publication date was imposed; Record content: Full record.			

Using “OR” between each term means the search returned documents containing any of these terms or phrases. The phrase between parenthesis, i.e., (Romania AND “environmental protection”), ensures that both terms must appear in the search results. This means that the search engine or database returned documents where “environmental protection” and “Romania” were mentioned together. The “AND” operator narrowed the search results by requiring both conditions to be met, ensuring that the results were relevant to environmental perceptions, specifically in the context of Romania. The “∗” operator was used to find different forms of a word that may include prefixes or suffixes. Specifically, the Boolean search found documents that contained any of the listed environmental terms (e.g., “environmental awareness” and “environmental values”) and also mentioned Romania (and sometimes “environmental protection”).

The documents were exported to the Zotero reference manager, and after removing the duplicates, 276 documents were retained for analysis. The keywords networks and clustering keywords were created with VOSviewer, version 1.6.20 [66], a free program. To perform the analysis, we opted for full counting as a counting method. This method considers the actual frequency of term occurrences within each document. If a term appears multiple times, each occurrence is counted. Full counting was considered more informative because we wanted to understand whether terms were related and also how strongly they were related within the literature (i.e., heavily discussed topics). The minimum of occurrences of a term was selected at 5, and out of the 8464 terms, 550 words met the threshold. For each of these 550 terms, VOSviewer calculated a relevance score. Based on this score, the most relevant terms were selected. The default choice was to select the first 60 % of the most relevant terms [66].

## 3 Results

### 3.1 Results from the qualitative research

In this section, we presented the data collected through interviews, highlighting participants' perceptions and behaviors regarding the environmental state in Romania. We used elements from the empathy map to structure and interpret this information. First, we detailed the coding method used and the illustrative quotes (Table A2). Subsequently, we synthesized the main ideas identified for each component of the empathy map, providing an overview of participants’ perceptions (Table 2). The aggregated empathy map synthesized common themes observed across participants and served as a step in their characterization.

**Table 2:** Aggregated information for the empathy map.

Question category according to the empathy map	Key Insights
***Think & Feel*** **WHAT DO I FEEL AND THINK ABOUT THE ENVIRONMENT?** What are my environmental values? What are my environmental needs?	**Values:** • Environmental protection as balance: Participants emphasized the importance of environmental protection in maintaining a balance between natural elements and human activities; • Human-Nature harmony: There was a recurring theme of empathy toward nature and the belief that a healthy environment contributes to human well-being and harmony; • Individual responsibility: There was a strong belief in personal responsibility for environmental actions and their impact, contrasting with the impact of larger entities like corporations; • Contributions to conservation: Participants expressed a belief that individual actions, no matter how small, contribute to environmental preservation and protection; • Personal commitment: Personal commitment to environmental protection, often originating from educational background and personal beliefs, was highlighted. **Influence on behavior:** • Proactive consumption: Participants mentioned behaviors such as consuming local products, reducing energy waste, and maximizing the use of resources to minimize environmental impact; • Policy advocacy: Some participants stressed the importance of involving scientists and the private sector in environmental policy development. **Needs and concrete measures:** • Air and green spaces: Improvement of air quality and availability of green spaces were highlighted as critical needs; • Public education and legislation: Participants emphasized the need for public education on environmental issues and better enforcement of environmental legislation; • Waste management and recycling: Effective waste management strategies, including selective recycling and reducing consumption, were considered essential. **The three main environmental problems in Romania perceived by participants were:** water and air pollution (85 % of the participants), deforestation (76 % of participants), and poor waste management (50 % of respondents)
***See*** **WHAT ENVIRONMENTAL PATTERNS BEHAVIORS DO I SEE?** What are the patterns of behavior around me?	**Behavioral patterns:** • Positive environmental behaviors: Proactive actions such as waste collection, tree planting, and care for green spaces were highlighted; Negative environmental behaviors: Detrimental behaviors such as littering or improper waste disposal. **External influence:** • Infrastructure limitations, and economic factors hidered participation in environmental programs; • Societal indifference and lack of interest toward environmental laws and regulations; **Personal determinants:** • Time constraints; • Poverty and basic survival needs can overshadow environmental concerns; • Personal psychological well-being can motivate or hinder environmental actions.
***Say & Do*** **WHAT DO I SAY AND DO ABOUT ENVIRONMENTAL PROTECTION?** What is my behavior toward the environment?	**Personal behavior:** • Engagement in environmental activities: Participants were actively involved in pro-environmental activities such as tree planting, recycling, separate waste collection, and caring for green spaces. **Obstacles:** • Infrastructure and facilities: The lack of infrastructure for efficient waste management was cited as a significant obstacle; • Convenience, economic, and time constraints hindered environmentally friendly actions; • Perceived lack of involvement from authorities and bureaucratic hurdles were obstacles; • Some expressed pessimism about making a difference due to perceived systemic issues. **Motivations:** • Health and well-being: Concern for personal and family health due to environmental degradation motivated participants; • Desire to live in a clean environment for the well-being of future generations; • Common good and conscience: Motivation rooted in common sense and conscience; • Desire to change the world for the better and maintain ecological balance; • Financial and incentive-based rewards: Monetary rewards were seen as potential motivators for eco-friendly behavior; • Aesthetic and emotional connection: Aesthetic appreciation of natural beauty motivates behavior; • Media coverage and public awareness also played a role in encouraging environmental actions; • Policy and coercive measures: Recognition that coercive measures might be necessary to achieve desired environmental outcomes.
***Hear*** **WHAT DO I HEAR ABOUT THE STATE OF THE ENVIRONMENT?** What are my sources of information? How do my family, friends, colleagues, etc. influence me?	**Sources of influence:** 47 % of the participants used conventional media, television, and the Internet as primary sources of information, followed by social networks (23 %). **Influence of peers**: • People adopted environmental protection behaviors when they observed others doing so. Examples set by peers, family members (like parents and spouse/partner), and social norms (such as those learned at school) shaped individual behaviors toward environmental Protection; • Success of behavior adoption: Personal experiences of adopting new behaviors, such as waste sorting, after seeing positive examples from others, demonstrated the effectiveness of peer influence and personal initiative in environmental actions. **Impact of cultural and environmental contexts:** • Environmental behaviors can vary based on cultural and environmental contexts. For example, behaviors in Romania might differ from those in Germany, indicating that the local context influences actions related to environmental protection.

**Fig. 2:**
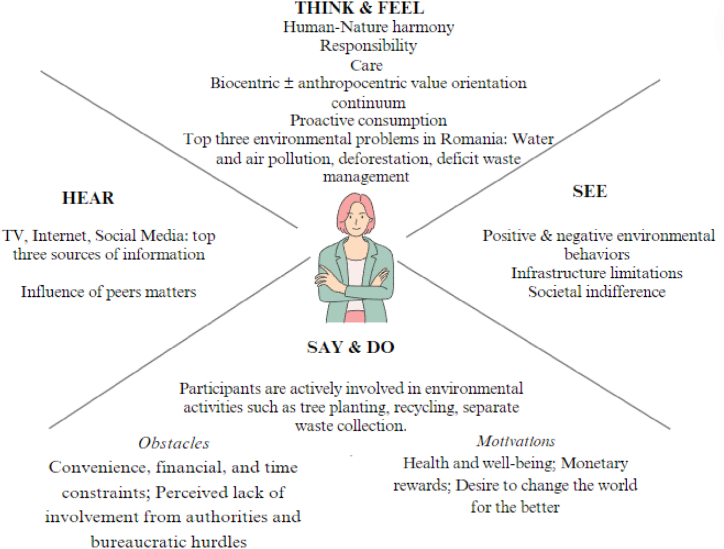
Empathy map: the main themes observed for each investigated area.

The aggregated empathy map (Fig. 2) synthesized common themes observed across participants, which helped in the respondents’ characterization.

### 3.2 Results from the quantitative research

#### 3.2.1 Visualization of the network

**Fig. 3:**
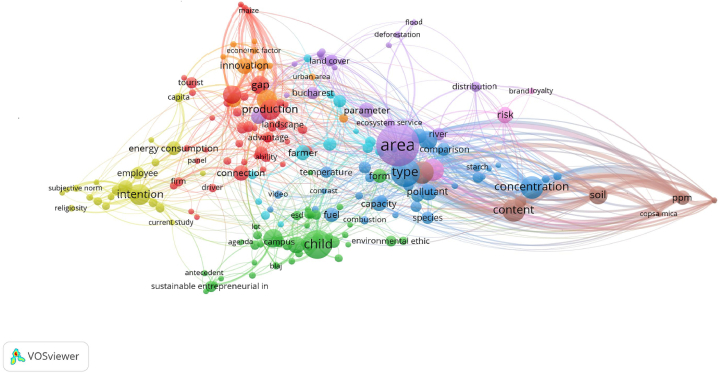
Map of keywords network visualization using VOSviewer software. Note: “esd” refers to education for sustainable development; “ppm” refers to parts per million.

Fig. 3 provides a network visualization of keywords clusters from the 276 selected documents. The visualization includes nine clusters, with each cluster representing a group of related keywords and distinguished by a unique color [66]. Additionally, the visualization features nodes and links, illustrating the connections between keywords. Each node represents a keyword, and the links between the keywords show the relationships or co-occurrences between these keywords. The strength and frequency of these relationships are indicated by the thickness and color intensity of the links [66]. Larger (i.e., font size) labels indicate keywords that occur more frequently within the analyzed text corpus.

Each cluster in the network represents a different thematic focus within the literature on environmental perceptions in Romania. The green cluster (no. 1; it includes 44 terms) that we named “Behavioral intentions and energy consumption” contains terms like intention, energy consumption, subjective norm, and sustainable entrepreneurial. We considered the keywords to suggest an interest in how personal beliefs and social norms drive sustainable behavior, particularly in entrepreneurial and energy consumption contexts. The red cluster (no. 2, it has 41 words), named “Economic and production dynamics”, has keywords like production, innovation, tourist, green marketing, economic factor, and gap. It is a cluster centered on the economic aspects of environmental sustainability, focusing on production processes, innovation, and tourism. The yellow cluster (no. 3, with 36 terms), “Energy, norms, and personal intention” has keywords such as energy consumption, intention, employee, self, and subjective norm. This cluster emphasizes the relationship between energy consumption and personal intentions within a social context. The dark blue cluster (no. 4, with 22 terms), “Environmental science and ecosystem services” is created around terms such as area, type, concentration, soil, ecosystem service, and parameter. It refers to aspects such as pollution concentration, soil health, and the overall capacity of ecosystems to provide services. The terms indicate a strong focus on the scientific and technical dimensions of environmental studies in Romania. In the purple cluster (no. 5, it includes 20 terms), “Socio-economic and environmental risk”, terms like risk, flood, and deforestation indicate studies on environmental degradation and natural disasters. At the same time, “poverty” and “distribution” suggest a focus on the socio-economic factors that influence or exacerbate these environmental risks. The intersection of agriculture, land use, and urbanization is highlighted in the orange cluster (no. 6, it has 14 terms), named “Agricultural and land-use practices”. It likely includes studies on sustainable agriculture and the impact of land-use changes on ecological balance. The light blue cluster (no.7, with 12 terms), “Urbanization and land cover”, focuses on urban areas, particularly Bucharest (Romania’s capital), and how urbanization impacts land cover and ecosystem services. The brown cluster (no. 8, it contains 7 terms), “Pollution and environmental measurement”, is created around terms like a pollutant, ppm, soil, concentration, and capacity. It refers to the measurement of pollution and its impact on the environment, focusing on quantifying environmental pollution, particularly in soils. The pink cluster (no. 9, with 3 terms), “Environmental ethics and sustainability”, is a smaller one, and it appears to focus on the ethical dimensions of environmental sustainability, including how consumer behavior is influenced by environmental ethics and how businesses use green marketing to appeal to environmentally conscious consumers.

There are dominant themes (large nodes), such as “area” (belonging to the purple cluster, “Socio-economic and environmental risk”), with a total strength of 1297, 121 links and 101 occurrences, and “type” (belonging to the dark blue cluster, “Environmental science and ecosystem services”), with a total strength of 1042, with 90 links and 56 occurrences. These appear as the most prominent nodes, suggesting that the spatial dimension (geographical or ecological areas) and the classification or types of studies or environments are central themes in the selected literature. This could indicate that much of the research focuses on specific regions within Romania or types of environmental contexts. Also, other prominent nodes are “concentration” (belonging to the dark blue cluster, “Environmental science and ecosystem services”) and “content” (belonging to the brown cluster, “Pollution and environmental measurement”). These terms also appear frequently, possibly related to studies focusing on pollution concentration or the content of environmental programs or policies in Romania. There are also less represented themes. One refers to health-related terms. The search strategy included terms related to “personal health and well-being”, but it seemed they did not generate significant returns, as they were absent or minimally connected in the network.

Additionally, we observed the existence of interconnected themes. The lines connecting the clusters indicate that there are shared themes or overlapping areas of research. For example, “land cover” and “urban area” link environmental science with spatial studies and urban planning, showing the interplay between ecological and urban development issues in Romania.

#### 3.2.2 Overlay visualization

**Fig. 4:**
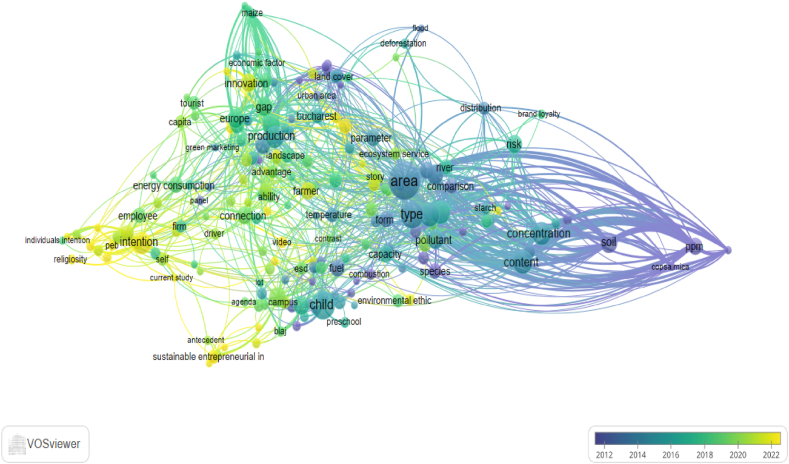
The map of overlay visualization of keywords co-occurrence based on average publications per year using VOSviewer software. Note: “esd” refers to education for sustainable development; “peb” refers to pro-environmental behavior; “ppm” refers to parts per million.

Fig. 4 offers another network visualization but includes an overlay of temporal data, likely representing changes or trends over time. What differs from Fig. 2 is the temporal overlay, the gradient color bar at the bottom right. The gradient ranges from 2012 to 2022 and indicates the time dimension of the data. Keywords and links colored closer to yellow are more recent, suggesting current or emerging trends. Those closer to blue are older and include foundational concepts and methods that have been studied longer.

The VOSviewer visualization in Fig. 4 reveals a dynamic evolution in environmental research in Romania, with earlier studies (in purple) primarily focused on foundational topics such as environmental science and pollution measurement. These early studies laid the groundwork by examining key environmental parameters, the distribution of risks such as deforestation and floods, and the overall capacity of ecosystems to sustain human activities. As the field matured, the research expanded to cover land use, agricultural practices, and the intersection of these with urbanization, reflecting a sustained interest in how land management and economic activities impact environmental sustainability.

In recent years (indicated in yellow), the focus has changed toward understanding behavioral intentions, energy consumption, and the role of social norms in promoting sustainable practices. There is an emphasis on sustainable entrepreneurship, highlighting the increasing recognition of businesses’ role in addressing environmental challenges. This trend suggests a broader shift toward applied research that seeks to influence individual and collective behaviors in ways that support sustainability goals.

#### 3.2.3 Density visualization

**Fig. 5:**
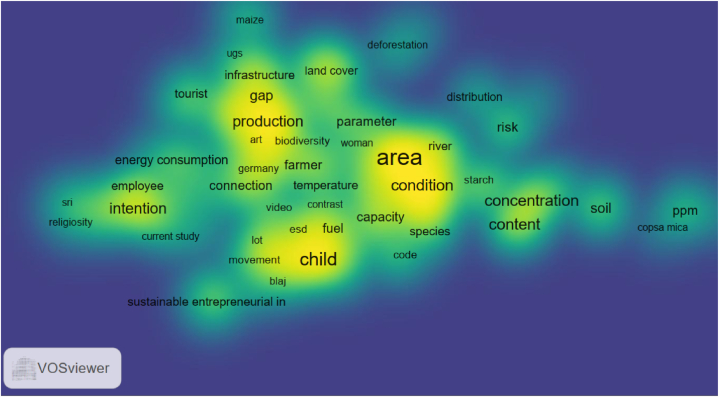
The map of density visualization of the keywords using VOSviewer software. Note: “esd” refers to education for sustainable development; “sri” refers to socially responsible investment; “ugs” means urban green spaces; “ppm” refers to parts per million; “blaj”, “copsa mica” are names of a city.

Fig. 5 shows the intensity of the research focus in different terms related to environmental perceptions in Romania. In this visualization, the colors range from blue (low density) to green/yellow (high density), with the most intense areas indicating the highest concentration of research.

The most intense yellow areas on the map are centered around the terms, e.g., “area”, “production”, and “intention”. These terms represent the focal points of research in the selected literature and suggest a significant research focus on spatial dimensions and classifications within environmental studies. This could include studies on specific geographic areas in Romania and different types of environmental contexts or classifications of environmental issues. Additionally, “production” strongly focuses on the relationship between environmental sustainability and production processes, likely reflecting concerns about how industrial and agricultural production impact the environment. The presence of “intention” suggests substantial research interest in understanding the behavioral intentions behind environmental actions, possibly related to how people plan to engage in pro-environmental behaviors.

Moderate-density areas (in green) are supported by terms like “energy consumption”, “species”, “innovation”, or “child”. For example, “child” may focus on environmental education or awareness among children, possibly examining how early education influences future environmental behavior. Low-density areas (in blue) contain terms such as “distribution”, and “deforestation”. These terms, although critical, appear to have a narrower focus in the literature, possibly indicating specialized studies or still underexplored areas.

## 4 Discussion

### 4.1 Discussion of the qualitative and quantitative results

The findings of the empathy map revealed a complex interplay between personal values, observed behaviors, and external influences shaping people’s perceptions of the environment in Romania (as illustrated in Table A2). Participants expressed a deep commitment to environmental protection, driven by a belief in maintaining a balance between human activities and natural elements. This belief manifested in values such as human-nature harmony, individual responsibility, and care for nature, where personal actions were considered critical contributors to restoration and conservation efforts [67]. Because values were frequently used to explain how we made new decisions, clear communication of these findings could guide practitioners in enhancing the socio-economic and ecological benefits of pro-environmental efforts, improving governance legitimacy, and increasing the social acceptability of environmental management actions [39,68]. Moreover, the absence of responses within the biocentric value orientation suggested a prevalent belief in balancing environmental protection with human needs rather than prioritizing nature independently of human interests. Despite some participants expressing pessimism due to systemic issues, the collective insights underscored a biocentric-anthropocentric value orientation continuum [69]. Thus, reciprocal harmony generated moral obligations toward nature, enabling humanity and nature to flourish together [70]. Research has often used the concept of the biocentric-anthropocentric value orientation continuum to explore why people value nature in specific ways and how these values have influenced environmental attitudes and behaviors [64,[71], [72], [73]].

We observed that the term “values” appeared recurrently throughout the responses in the entire interview guide, mainly related to behaviors, expressions of concern, and optimism about human impacts on the environment (see Table A2 for extracts from the interviews). The interviewed people appeared to adopt often what Dietz et al. [74] considered “values”. These values were most commonly related to self-reported behaviors (such as sorting waste), behavioral intentions (like participating in reforestation campaigns), or other actions that showed concern for the environment. This approach was partially encompassed in Schwartz and Bilsky’s work [75] “values are (a) concepts or beliefs, (b) about desirable end states or behaviors, (c) that transcend specific situations, (d) guide selection or evaluation of behavior and events, and (e) are ordered by relative importance”. According to Dietz et al. [74], shifts in values were believed to drive changes in decisions, which subsequently influenced behavior. Numerous studies associated values with pro-environmental attitudes. Research (e.g., Refs. [7,76,77]) helped us understand how values might impact actions by identifying pro-environmentally individual behaviors, such as green consumer choices, energy consumption reduction, climate change adaptation, and active and passive political support for environmental initiatives.

Participants frequently mentioned proactive habits, such as buying local products and reducing energy waste, as practical expressions of their environmental values (see Table A2 for extracts from the interviews). As Richardson et al. [78] observed, basic activities strengthened our connection with nature and were essential in inspiring efforts to care for nature. In addition, there is a call for greater public education and stricter enforcement of environmental legislation to address critical needs such as improving air and water quality, deforestation, and effective waste management. In Brazil, a country that struggles with the deforestation phenomenon, improvement of environmental legislation, particularly between 2005 and 2012, and policies like the Rural Environmental Registry (CAR) contributed to the decrease in deforestation rates [79]. However, despite this personal commitment, participants in our study acknowledged significant barriers, including infrastructural limitations, time, and financial constraints, which often impeded broader participation in pro-environmental activities. Moreover, when addressing financial constraints in environmental protection, proactive measures could be more cost-effective than reactive responses [80]. This approach could include fostering broader stakeholder participation, which could bring diverse funding sources and community support for conservation projects. Much research highlights a complex and mixed set of results regarding how financial circumstances influence people’s involvement in pro-environmental behaviors [81,82]. Additionally, using efficient methods like early detection systems for species decline could complement traditional, resource-intensive studies, reducing costs [80].

Behavioral patterns observed by participants in their peers indicated a dichotomy between positive actions, such as waste collection and tree planting, and negative behaviors, such as littering, influenced by external factors, such as economic constraints and lack of infrastructure. It is important to improve coordination between public and private actors to address systemic barriers like lack of infrastructure and financial constraints, especially in funding environmental initiatives [83,84]. Encouraging co-financing from private actors, such as real estate developers or residents who benefit from environmental projects, can help bridge funding gaps. Additionally, integrating the benefits of nature-based solutions into valuation and accounting methods could attract more investment by highlighting their economic value [83], making it easier to justify and secure funding for such projects.

This need for tailored solutions also reflected the cultural and economic contexts that shape environmental behaviors differently across regions. For instance, the perceived differences in environmental actions between Romania and other countries highlighted the role of these contexts. This observation aligned with other research findings, such as those of Miller et al. [85], who investigated the connections between environmental attitudes, efficacy, and pro-environmental behaviors in 11 countries. They concluded that cultural, social, and political contexts distinctly shaped individuals’ environmental attitudes and engagement in pro-environmental activities. Thus, addressing environmental behaviors effectively requires not only overcoming economic and infrastructural challenges but also considering the cultural context of each region.

We found that mass media (mainly television and the Internet) and social media are the two most used sources to inform people about environmental issues, with peer influence and social norms significantly impacting behavior adoption. Exposure to environmental information on social media platforms was widely shown to influence individuals’ intention to participate in pro-environmental behavior [86,87]. Moreover, the role of social capital, including social trust, social norms, and social networks, was proven to promote pro-environmental behavior [88].

Considering all the above, a significant result from the qualitative data, also observed in Dietz et al. [74] study, was that values played a crucial role in shaping individual actions. These actions, in turn, substantially influenced both personal and collective behaviors toward the environment.

This connection between values and behavior is further reflected in the network visualization that provided a comprehensive overview of key themes and relationships in the literature on environmental perceptions in Romania. The bibliometric method of the analysis of keywords co-occurrence indicated considerable attention to economic factors, sustainability in business practices, and the intersection between environmental science and urban or regional studies. The results could suggest a robust interdisciplinary approach with opportunities to expand research into less explored areas, such as health impacts and motivations for personal behavior. Therefore, the less-represented topics, such as health-related terms, could suggest a gap in the literature regarding the intersection of environmental protection and personal health in Romania, or it might indicate that such topics were discussed under different terminologies not captured in our initial keyword selection. The environment-health nexus encompassed a broad range of environmental determinants, including digital, psychosocial, political, socioeconomic, and cultural factors, to understand the intertwined relationship between environmental changes and human health [89]. The environment-health nexus operates within a holistic framework, and it requires collaboration at professional and organizational levels for a healthy nation [90]. The focus had shifted from foundational environmental science and pollution studies to more contemporary concerns with behavioral change, energy consumption, and sustainable entrepreneurship. Considering how the focus area had changed over time, the interpretation of the overlay map underscored the growing complexity of environmental challenges and the need for integrated approaches that combine scientific understanding with practical, informed solutions. As per Burke et al. [91], there is a need for a systems-based approach using transdisciplinary and systems science to effectively address complex environmental challenges and protect the environment.

### 4.2 Cross-analysis of quantitative and qualitative data

**Fig. 6:**
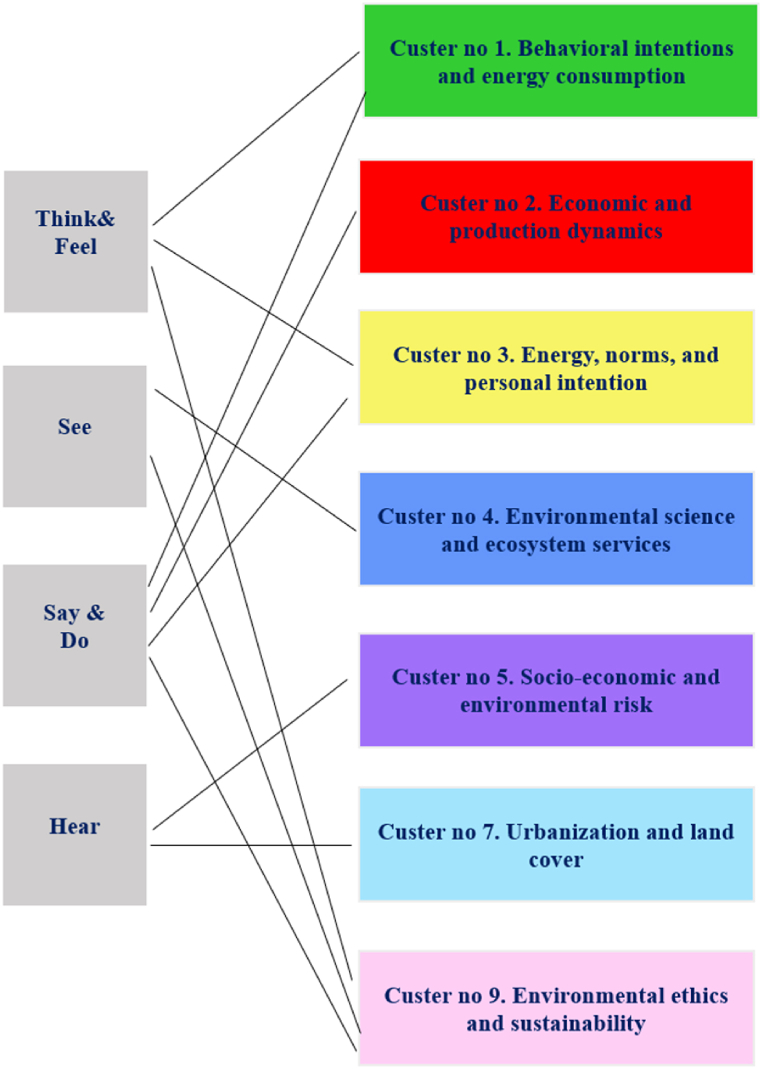
Cross-analysis visualization: qualitative themes and quantitative clusters.

The most prominent or representative clusters that aligned closely with the qualitative themes identified in the empathy map are discussed in the following and illustrated in Fig. 6. The intention was to highlight key cross-findings between the qualitative and quantitative analyses by focusing on the most significant or thematically relevant clusters.

Within the “Think & Feel” component, participants expressed a strong sense of individual responsibility and personal commitment to environmental protection. This perspective aligned with the focus of the green cluster on behavioral intentions and energy consumption (Fig. 3), suggesting that the qualitative emphasis on personal responsibility was reflected in the focus of the Romanian literature on how individual intentions drove sustainable behavior. In contrast, a study by Jia et al. [92] involving 164 mid-sized American university students identified concern for other species and disapproval of environmentally irresponsible behavior as central motivations among activists. Unlike these broader motivations related to guardianship, our study emphasized how participants’ sense of responsibility was more directly linked to their own actions in fostering change. This difference highlights a more individualistic approach in our findings, focused on the impact of personal commitment in driving sustainable practices.

The focus on personal agency aligned with the strong presence of terms like “intention” and “energy consumption” found in the keyword co-occurrence analysis. This correspondence suggests that themes around personal and social norms, particularly regarding energy use, were central in the context of Romanian scientific literature. This trend is present in other research focused on other countries. Belaïd and Flambard [93] investigated how social norms and informational tools affect people’s preferences for energy efficiency in building design and construction. Energy, norms, and personal intention (cluster no. 3, yellow) related to the qualitative insights around social norms, personal intentions, and energy-related behaviors, which were key in both the “Think & Feel” and “Say & Do” components. This finding was consistent with similar interests in the topic. Fornara et al. [94] showed that moral norms and informational influence were the strongest predictors of household intention to use renewable energy devices, mediated by social norms. In addition, values and other concepts related to human-nature harmony were central themes in the thematic analysis and aligned well with the focus of cluster no. 9 (pink), “Environmental ethics and sustainability”. Participants' focus on environmental protection as a balance between natural elements and human activities is closely related to the ethical considerations underlying sustainability. Similarly, Zheng and Dai [95] argued that environmental ethics was crucial for understanding human-environment interactions and fostering sustainable development through actions such as education, regulation, and inspiration. This cluster’s focus on the ethical aspects of sustainability resonated with participants' values and beliefs, highlighting how moral norms and ethics drove individual and collective efforts toward environmental protection.

In the “See” component of the empathy map, participants noted the importance of observing positive environmental behaviors and the need for improvements in areas such as air quality and green spaces. Similarly, global scientific literature [96,97] reported on interventions that emphasized how common pro-environmental behaviors triggered positive shifts in the audience’s behavior, including those related to energy use [98]. The findings from the “See” component are reflected in the blue cluster focus on scientific and technical dimensions of environmental studies, such as soil health and ecosystem services. Terms such as “area”, “type”, and “concentration” could suggest a detailed examination of specific environmental parameters, which resonated with participants' emphasis on observable environmental conditions and the need for concrete measures in the literature.

The “Say & Do” component showed that the participants identified obstacles such as economic constraints and infrastructure limitations as significant barriers to engaging in pro-environmental behavior. This corresponded to the focus of the red cluster (no. 2) on economic factors and production processes, indicating that the economic aspects highlighted in the thematic analysis are well-represented in the literature. The presence of terms such as “economic factor” and “production” in the red cluster aligned with the respondents’ concerns about the economic challenges of environmental protection, indicating a close connection between qualitative insights and the thematic focus of the literature. Similarly, systemic barriers (e.g., lack of infrastructure and financial constraints) were pointed to be so significant that shifting behavior became very expensive, diminishing the role of personal motivation in achieving positive environmental results. For example, in a review by Steg and Vlek [99], authors highlighted that behavior was shaped by a broader set of influences, with external factors playing a crucial role in enabling or restricting pro-environmental actions. For instance, the presence of recycling options, the efficiency of public transportation, the availability of sustainable products, and pricing policies could greatly impact how easily people adopt eco-friendly habits. Other research demonstrated how economic costs and infrastructural challenges could create asymmetries between intentions and actual pro-environmental behavior, particularly when the necessary systems or resources were not available. Whitmarsh [100], who investigated energy-saving behaviors in the UK, found that transport infrastructure and urban design strongly influenced travel choices. People in rural areas were more likely to rely on driving due to limited alternative options.

The “Hear” component highlighted concerns about environmental risks, including deforestation and floods, which participants heard about through media and other sources. This was consistent with the focus of the purple cluster (no. 5) on socio-economic and environmental risks, such as flood risk. The analysis of keywords co-occurrence that identified terms such as “risk”, “flood”, and “poverty” indicated the themes of environmental risk and socio-economic factors. This finding underscored the alignment between participants' perceptions and the emphasis found in the existing literature. This connection was further supported by Lorenzoni and Pidgeon [101] who explored how media and other sources shaped public awareness and concern about environmental risks, such as deforestation and flooding. Their work discussed the significant role of communication channels in influencing how individuals perceived and responded to those environmental challenges.

Participants mentioned in the “See” and “Hear” components their awareness of urban environmental challenges, such as the impact of urbanization on green spaces and pollution. This awareness aligned with findings from Kabisch et al. [102], who reviewed the impact of urbanization on green spaces and how people perceived and responded to these changes. Their review highlighted similar concerns about the loss of green areas due to urban development and the growing pollution problems in urban settings, reflecting the same themes mentioned by the participants in the present study. This alignment was further evident in the light blue cluster on urbanization and land cover from the keyword co-occurrence analysis.

Clusters 6 (orange) and 8 (brown), namely “Agricultural and land-use practices” and “Pollution and environmental measurement”, were less well-represented in the thematic analysis, probably because the qualitative research focused more on personal perceptions, behaviors, and social influences on environmental protection than on technical or sector-specific aspects.

*Limitations and future research directions*. Qualitative research on local perceptions reveals certain limitations, including the possibility that these perceptions may be inherently subjective and may not always accurately represent the actual conditions of built and natural environments [103,104]. This subjectivity can complicate efforts to use perceptions as reliable indicators of environmental outcomes or to determine the causal impact of pro-environmental initiatives. Other challenges include the potential for biases in self-reported data, as individuals may report perceptions influenced by social desirability or external pressures. The context-specific nature of qualitative insights can also limit their generalizability, which poses challenges for applying findings across different settings or scales. Additionally, qualitative research tends to be time-consuming and resource-intensive, which can restrict the number of participants included in the study and limit the overall scope of data collection. Another limitation is that the relationship between values and behavior can depend on the specific types of values being examined. Thus, future research should include a wide range of environmental values and explore longitudinal designs to assess changes in values and behaviors over time.

Referring to the analysis of keywords co-occurrence, despite many new research papers being added to the WoS daily, only a subset, in English, is indexed in the core database, often leading to the exclusion or neglect of many non-English language articles [65]. This language bias may result in an incomplete picture of global environmental research trends, as studies published in Romanian (for example) can contain relevant findings that remain unaccounted for. Additionally, selecting specific keywords can introduce bias, as it can exclude relevant studies that use different terminology. Moreover, reliance on particular databases like WOS can limit the scope of the analysis, as these databases do not cover all relevant publications, particularly those in emerging or interdisciplinary fields. The ever-evolving nature of environmental research means that new keywords and themes may emerge over time, which could result in a lag in recognizing and incorporating these developments into the analysis.

## 5 Conclusions

The study argued that understanding perceptions was critical to fostering a collective commitment to environmental stewardship and sustainability. By exploring these complex attitudes, needs, values, and behaviors, we could better understand the motivational drivers that encourage pro-environmental actions among Romanians. The empathy map revealed that the Romanians interviewed were deeply committed to environmental protection, driven by a desire to balance human activities with natural preservation. They held strong values of human-nature harmony and individual responsibility, viewing personal actions as essential pro-environmental efforts. Despite systemic challenges, their collective outlook was shaped by a biocentric-anthropocentric value orientation, emphasizing respect and care for nature alongside human needs.

Using bibliometric analysis, we could validate the themes identified in the qualitative phase, checking whether the personal perceptions uncovered through the empathy map aligned with or diverged from the more significant trends in existing research. For instance, participants’ emphasis on economic constraints and infrastructural challenges was mirrored by a strong focus on economic factors in the keyword clusters. Consequently, the analysis of keywords co-occurrence helped us to identify gaps and trends in research that aligned with the themes from the interviews, thereby providing a more comprehensive understanding of the discourse surrounding environmental perceptions in Romania.

These findings offer several practical implications for policymakers, particularly in designing policies that align with the values and motivations of the public. For instance, the emphasis on personal responsibility and community-driven environmental efforts suggests that policies encouraging local environmental initiatives, such as community-based recycling programs and incentives for purchasing local products, can resonate well with the public. Moreover, the identified barriers, such as economic constraints and infrastructural challenges, indicate a need for targeted investments in infrastructure, like improved waste management systems and accessible green spaces. Such investments could enable a more extensive participation in pro-environmental behaviors. The insights from the empathy map can also guide public awareness campaigns that emphasize the connection between individual actions and broader environmental outcomes, thus leveraging the existing solid values of human-nature harmony to promote sustainable behaviors. Additionally, these findings can inform educational programs that address the knowledge gaps identified in our study, helping build a more informed citizenry that can support environmental policies.

## CRediT authorship contribution statement

**Ruxandra Malina Petrescu-Mag:** Writing – review & editing, Writing – original draft, Visualization, Validation, Supervision, Software, Methodology, Investigation (Bibliometric analysis), Formal analysis, Data curation, Conceptualization. **Adrian Ivan:** Writing – original draft, Conceptualization. **Cornel Pantelimon:** Writing – original draft, Investigation (Interviews), Conceptualization. **Dacinia Crina Petrescu:** Writing – review & editing, Writing – original draft, Methodology, Formal analysis, Data curation, Conceptualization.

## Ethics and consent

The Research ethics approval was issued by The Scientific Council of the Babes-Bolyai University of Cluj-Napoca, no 1148/2/26.01.2024.

## Data and code availability

Data will be made available on request.

## Declaration of competing interest

The authors declare that they have no known competing financial interests or personal relationships that could have appeared to influence the work reported in this paper.

## Figures and Tables

**Table A1:** The script for the one-to-one interview

Question category according to the empathy map	Interview question
***Think & Feel*** **WHAT DO I FEEL AND THINK ABOUT THE ENVIRONMENT?** What are my environmental values? What are my environmental needs?	*Values:* What does the environment and its protection mean to you? What are your central values or beliefs related to protecting the environment? How have these values changed over time? If they have changed, why? Can you give us a concrete example of how you acted under the influence of these values? How do environmental values influence consumption, lifestyle, and behavior decisions? Can you give us some examples? What needs do you feel the environment should fulfill for you? How would the environment improve or change to meet these needs? *Needs:* What concrete steps should ordinary people or policy-makers take to meet your environmental needs? How do you see yourself contributing to these efforts? Can you list the top three most severe environmental problems in Romania?
***See*** **WHAT ENVIRONMENTAL PATTERNS BEHAVIORS DO I SEE?** What are the patterns of behavior around me?	*Third-party environmental behavior:* Have you noticed specific behavior patterns (positive and negative) (please mention them) related to the environment that are common among people in your community or workplace? What motivates people to adopt certain friendly behaviors with the environment? Have you noticed situations where people give up environmentally friendly behaviors because of external factors?
***Say & Do*** **WHAT DO I SAY AND DO ABOUT ENVIRONMENTAL PROTECTION?** What is my behavior toward the environment?	*Own behavior:* What initiatives or actions have you taken to help protect the environment? *Obstacles* What are your main obstacles or difficulties in adopting environmentally friendly practices on a daily basis? *Motivations* What motivates you to participate in environmental actions or initiatives?
***Hear*** **WHAT DO I HEAR ABOUT THE STATE OF THE ENVIRONMENT?** What are my sources of information? How do my family, friends, colleagues, etc., influence me?	What are your primary sources of information for making decisions related to environmentally friendly behavior? Have you ever taken an action related to environmental protection just because you saw others doing the same thing?
**Demographic variables**	
Age	Open question
Gender	a) F b) M c) Other
Education	a) 8 classes b)12 classes c) university
Average monthly income for the whole family	a) maximum 1000 lei/month b) 1001–3000 lei/month c) 3001–5000 lei/month d) 5001–7000 lei/month e) more than 7001 lei
Residence	a) Urban b) Rural
I have children	a) Yes b) No
I suffer from chronic diseases.	a) Yes b) No
I work in the field of environmental protection.	a) Yes b) No

**Table A2:** Question categories for the empathy map and coding methods with direct quotes

Question category according to the empathy map interviews ∗	Deductive codes (including the codes for the script interview questions, second column, Table A1)	Direct quotes (examples) associated with deductive coding	Inductive coding	Direct quotes (examples) associated with inductive coding
***Think & Feel*** **WHAT DO I FEEL AND THINK ABOUT THE ENVIRONMENT?** What are my environmental values? What are my environmental needs?	*For values:* The environment means nature and people; Respect for nature; Care for nature; Biocentric values; Anthropocentric values; Biocentric - anthropocentric value orientation continuum *For values influence on behavior:* Adopting pro-environmental behaviors. *For needs and concrete measures to take* Desired environmental improvements/changes; Collaboration/partnership. *For the top three environmental problems in Romania:* Waste management; Pollution (in general); Climate change; Deforestation.	*For values:* “For me, the environment means water, air, green spaces, flora and fauna, nature, and the environment. And even society. And its protection means ensuring a balance between them.” (P1)∗∗ “(…) such environmental values can be human-nature, ecological balance, and human well-being. Yes, they have changed over time, but not for the better, rather for the worse. And there was a reason: […] the education of people.” (P2) “All that was created naturally, not artificially, by humans, and therefore, from my point of view, environmental protection means safeguarding the biosphere of all areas where life exists, which involves fulfilling a moral obligation. Protecting humanity … The living environment must ensure the development and perpetuation of the human species under biosecurity conditions.” (P7) “(…) I am fully convinced that our health and well-being depend on a clean environment. The environment should be as close as possible to its natural state.” (P9) “A core value that I consider is empathy toward nature, if this can be considered a value. The word ‘value’ should also be equivalent to the environment because it is highly valuable to humans. (…) Among other values are human-nature harmony, well-being, and a healthy environment. All of these contribute to the harmony between humans and the environment. If nature is healthy and people are healthier, it leads to a better state and what they do for other people and at the same time for the environment.” (P14) “The environment is practically, as I imagine it, like the symbol of yin and yang. If we are yin, the environment is yang. Together with the environment, we form a whole, unified, and inseparable.” (P19) “Deforestation, air pollution, and the waste. You can see it along the roads, where waste is often dumped without following any rules.” (P23) “I believe that the relationship with the environment should be based on respect, that is, we must co-exist in harmony without any negative influences and repercussions as a result of our interaction.” (P24) *For values' influence on behavior:* “I prefer to consume local products, both because of the reduced emissions associated with logistics and packaging, and so on. Additionally, there’s the aspect of clothing you no longer wear in public, which has been slightly worn out and can be used for sports, at home, or household chores. I also take care not to waste energy, and so on. I think about being proactive because it’s okay not to consume energy, but how can I reduce consumption this way? Here, I refer to additional insulation for the home.” (P9) “(…) behave responsibly toward the environment in any situation: when we go out into the greenery, use a car, or are in the house to sort our waste, shopping, or as consumers, in general. Let’s leave what the Earth and the Universe offered us to future generations. Let’s consume as sparingly as possible.” (P3) “I can say that my consumption routine is controlled, or rather, I try to use everything fully, avoiding waste and using a very high percentage of any food or resource. This applies both to food and products, in general. I try to use a detergent until the last drop and consume a type of food until I finish it.” (P9) “I find it very important for scientists and the private sector to participate in the development of policies for environmental protection.” (P16) “I am also careful with energy consumption, ensuring not to waste it, and so on. I also think about acting proactively, in the sense that it’s good not to consume energy, but how can I consume even less in this way? I am referring to additional insulation for the house.” (P23) *For needs and concrete measures to take:* “Improving air quality in urban areas is one of the main needs. Also, ensuring sufficient green spaces for the city’s population and maintaining cleanliness.” (P2) “Ordinary people are less familiar with the legislation, but at least in local communities, authorities and municipalities should be responsible for popularizing and informing them about how to use wastewater and sort waste (…). Therefore, everything should be designed so that people can understand it, in the most accessible language possible. They should also be informed about legislative restrictions that could lead to fines if certain rules are not followed.” (P3) “So, the environment should provide us with the food we need, as well as fresh and clean air. And the water necessary for hydration that is not polluted.” (P8) “(…) to implement a better strategy than the current one for selective collection, controlled storage, and recycling of the resulting waste, as it is increasingly abundant. Consumption rates are high, and waste management is a major global issue. Every citizen should have a civic attitude at the population level, not necessarily waiting for fines, but acting in good faith toward environmental protection.” (P10) “Referring to concrete steps that ordinary people and I could take, I think about recycling, especially selective waste collection. Although it has been implemented in our country, I don’t think it works as well as it is claimed. Furthermore, walking more, perhaps reducing air travel, as well as maritime transportation, as these vehicles also pollute quite a lot.” (P15) “I only consume what I need, I adopt a balanced lifestyle, I try not to pollute, I try to sort the waste and dispose of it properly. I do not waste water or energy; I turn off the water and the lights.” (P28)	*For values:* Responsibility; Commitment. *For the top three environmental problems in Romania:* Lack of education; Lack of coherent environmental legislation; Industry impact on biodiversity.	*For values:* “(…) that’s why everything possible should be done through recycling, avoiding waste, and consuming responsibly, such as products from local producers or from one’s own production.” (P9)” “Each person must be responsible for their actions. If an individual recycles and the environment becomes clean, the individual has a certain impact, but if a company pollutes, it nullifies all of that individual’s effort.” (P24) “The belief that responsibility toward future generations and the belief that each individual can contribute to the conservation and protection of the environment through small yet consistent actions.” (P33) “The belief that I can make a difference. I was drawn to this topic and pursued the path to this field, starting from high school with a specialization in ecology and environmental protection and continuing through university and master’s studies in the same field.” (P29) *For the top three environmental problems in Romania:* “Education is an environmental issue. It starts with people’s formation, and that’s where problems begin.” (P9) “(…) the lack of coherent, applicable, and effective legislation, to promote a healthy environment.” (P16) “So basically, there is a disruption of biodiversity and the lives of animals and fish in the case of industrialization. Yes, industrialization means human intervention for certain benefits, to the detriment of living beings.” (P25)
***See*** **WHAT ENVIRONMENTAL PATTERNS BEHAVIORS DO I SEE?** What are the patterns of behavior around me?	*For the top three environmental problems in Romania:* Waste management; Pollution (in general); Climate change; Deforestation *For external influences*: Economic factors; Time constraints; Convenience; Lack of infrastructure	*For behavioral patterns:* “Positive behaviors – waste sorting, tree planting, flower planting, care for green spaces.” (P1) “In my community, where I live, the local authorities encourage the waste collection and sorting by components: glass, metal, paper, and cardboard.” (P7) “(…) most of the population does not maintain cleanliness, does not bother to deposit waste selectively.” (P10) “When I go to Tarnița Lake and see the dam filled with garbage, I don’t see the water's surface, just PET bottles. It upsets me when I see that some people are trying to improve the environment while others undermine it.” (P18) “Consumerism, which has a negative impact, but it seems to be gradually decreasing, at least from what I have observed. People have started to pay more attention to how much they consume and what they consume.” (P30) *For external influences*: “The limited facilities available for those participating in environmental protection programs reduce interest in existing initiatives. Additionally, implementing differentiated pricing based on environmental impact could prove to be costly.” (P7) “Yes, lack of health and money, but especially lack of time, are factors that have prevented truly protecting the environment.” (P14) “In my city, people don’t have much time; it’s very crowded. The thought of recycling bottles seems daunting to some, and some choose not to do it. I think time is the most used excuse for not recycling.” (P15) “Nobody has time to go, pick up some trash from the forests, dispose of it or collect it selectively.” (P31) “(…) when you have nothing to eat or nothing to heat with, you no longer care about the environment. I know a concrete example: you make a fire with whatever you find, bags of clothes bought for 20 lei, or burn anything to keep warm, including tires. Lack of money, poverty, would be a very, very important factor in this regard.” (P19)	*For external influences*: Indifference/Lack of interest toward legislation; Embarrassment; Psychological well-being.	*For external influences*: “I have also noticed indifference and lack of interest in current environmental norms and legislation.” (P2) “There are still people who feel not necessarily intimidated but ashamed when others judge them; and people do judge them, but probably not to the extent they think. It’s because of shame; that’s how it seems to me.” (P12) “If I am well and feel good, I do everything right. If I am anxious, I have family problems, if I am upset, then for three days, I did not feel like recycling. I give myself as an example. It all depends on us, on our psychological well-being.” (P18) “Too little interest from people, and probably negligence, which leads to a negative impact” (P22)
***Say & Do*** **WHAT DO I SAY AND DO ABOUT ENVIRONMENTAL PROTECTION?** What is my behavior toward the environment?	*For personal behavior:* Pro-environmental activities: Community involvement; Consumer choices; Lifestyle changes. *For obstacles:* Lack of Awareness; Cost/Budget constraints; Infrastructure/Accessibility; Time constraints. *For motivations:* Values; Environmental concern; Health and well-being; Concern for the self; Financial rewards.	*For personal behavior:* “I got involved in tree planting, care of green spaces, and waste sorting on a smaller scale.” (P1) “I try to buy the healthiest products, the healthiest foods possible, …, given that I work in education, I participated, last year, in Green School Week, during which various activities were carried out at school. Such activities encourage environmental protection to prevent negative outcomes.” (P5) “I tried with my friends to plant trees. We did a reforestation project together to help nature grow.” (P22) *For obstacles:* “It would be the lack of infrastructure and facilities that allow efficient waste management.” (P1) “Probably the lack of time, and this rush, do not leave you with time to take environmentally friendly actions.” (P6) *For motivations:* “I believe that people are primarily motivated by common sense.” (P1) “The environmental degradation that exists, observing how over time the air I breathe, along with my children and family, degrades. And effectively, the environment, the soil, the water all affect the health of my children, my family, and those close to us.” (P8) “The desire to change the world for the better.” (P11) “There is no specific motivation other than consciousness.” (P27) “Once again, their interest takes precedence over the common good. People fear for their health. They care about their health. They are convinced that a favorable, less altered environment can only bring them benefits.” (P17) “I believe that many people are motivated by monetary rewards. For example, a sum is received for each bottle if bottles are taken for recycling. This would encourage people to be more attentive because they receive something in return.” (P23) “Usually, financial motivation is the only aspect that would push people to change their behavior.” (P26) “Information. Education. Understanding the idea that the environment is a future resource that everyone needs” (P30)	*For obstacles:* No obstacles; Convenience; Lack of public authorities' support; Bureaucratic hurdle; Environmental fatalism. *For motivations:* Public/self-awareness; Care for the future generation; Aesthetic connection; Familiarity with the topic; Fear of coercive measures.	*For obstacles:* “I have no obstacles in getting involved in environmental protection.” (P1) “It is comfortable to call the courier to bring you something ordered at home. The comfort of our days uses a lot of resources that also affect the environment.” (P3) “A lack of involvement from the authorities.” (P11) “(…) from the person who holds a piece of paper in their hand because they can’t find where to throw it away, to the businessman who doesn’t get an environmental permit because he lacks a stamp, not because he doesn’t meet the conditions but because of a missing stamp. From infrastructure to public policies, these things can hit you and make you give up, at some point just giving up on being environmentally friendly.” (P16) “Perhaps my pessimistic belief that it doesn’t matter what we do anyway, that we have already exceeded the time frame in which we could have made a difference.” (P19) *For motivations:* “Public awareness of the natural ecological balance and its fragility can motivate them to adopt eco-friendly behaviors and habits.” (P2) “Self-awareness. Because I care about the environment and want to take care of it as much as I can.” (P14) “I want my children to live and have fun in a clean environment. My future children.” (P2) “I think most of us are motivated by thinking about the future of our children and the generations to come.” (P5) “The beauty of a place. The more beautiful a place is, the more motivated you are to keep it clean. Whether you go to the mountains or the sea, you want to enjoy a beautiful and clean environment. If you find a clean place, you don’t feel like littering and treat nature differently.” (P6) “The media coverage of environmental protection on online platforms such as Instagram, Facebook, TikTok, and others.” (P32) “Without coercive measures, we will not achieve the results we desire.” (P34)
***Hear*** **WHAT DO I HEAR ABOUT THE STATE OF THE ENVIRONMENT?** What are my sources of information? How do my family, friends, colleagues, etc., influence me?	*For sources of information:* Social Influence; Media Influence; Education. *For peer influence:* The power of example.	*For the peer influence:* “I believe that people adopt environmental protection behaviors both when they see them in others and when forced to adopt them. Small steps taken collectively by humanity are important examples.” (P13) “Usually, the power of an example is very useful. I’ve seen that people take more actions if they see others doing them first.” (P14) “Most of the time, the power of example prevails when we make certain decisions; what we see in others, we try to copy as something good, which are positive aspects. Similarly, information from various sources, such as family and school, are sources of information and contribute to influencing behaviors.” (P17) “For example, the first time I met someone involved in waste sorting was with my boyfriend’s parents, and obviously, I wanted to implement it with my own parents as well. I didn’t think it would be successful, but it was.” (P26) “Children tend to adopt environmentally friendly behavior when they see it in adults or when they are informed about it.” (P30)	*For sources of information:* Church. *For peer influence:* Country context as a source of influence	*For sources of information:* “Thirdly, I would refer to the church as a source of influence in making decisions related to environmentally friendly behavior.” (P21) “The country context people live in. For example, they may behave in a certain way in Romania and differently in Germany.” (P34)

Notes: ∗The questions included in the empathy map model of [45,46] are highlighted in capitalized fonts; the questions created by the authors to reflect the questions of the model are mentioned in italics; ∗∗P1, P2, …, P34 = The 1st, 2nd, …, 34th participant in the interviews.
